# Academic Framing as a Cause of Eco-Anxiety

**DOI:** 10.3390/epidemiologia4010006

**Published:** 2023-01-28

**Authors:** Arnaud Chiolero

**Affiliations:** 1 Population Health Laboratory (#PopHealthLab), University of Fribourg, 1700 Fribourg, Switzerland; achiolero@gmail.com; 2 School of Population and Global Health, McGill University, Montreal, QC H3A 1G1, Canada

Eco-anxiety, a highly mediatized emotion that is complex to characterize [1,2], seems to have reached epidemic proportions. Hence, as revealed by a recent large international survey including 10,000 persons aged 16 to 25 years, it was poignant to read that a large share of young people is desperate about the future of our planet [3]. Asked about their emotions, 67% of participants reported being sad, 51% helpless, and 61% powerless about climate change. Even more striking was that 56% considered humanity to be doomed and 39% reported being hesitant to have children. What are the public health implications of these observations? I argue here that researchers should be wary of how they can contribute to eco-anxiety by the way they frame their research and their narratives of the evidence [4,5].

**Framing**

It is legitimate that young people feel anxious about climate change because they are exposed to anxiogenic narratives about the impact of climate change with no way to cope [6]. Studies indicate that uncertainty and unpredictability but also uncontrollability are important drivers of eco-anxiety [2]. Hence, asked about governmental action, 64% of participants in this international survey considered that governments did not take their concerns seriously enough, 62% that they cannot be trusted, and 65% that they failed young people across the world [3]. It is striking, however, to note that the survey did not include questions about potential solutions to address the climate challenge; the authors have explored eco-anxiety and to what extent governments should be blamed for their inaction but did not assess responders’ views on what could be done or if they considered themselves as actors to solve the problem, which could reveal a framing bias. 

Framing is to select some aspects of perceived reality and make them more salient [7]. To have health issues put on the agenda, articulation of a scientifically and socially credible threat is necessary [8], and framing climate change as a human health issue is sound to influence policymakers [9]. One expects, however, researchers studying eco-anxiety to embrace a comprehensive, apolitical, and balanced perspective. Actually, while eco-anxiety can paralyze, it can be also seen as a form of “practical anxiety”, that is, an emotion leading to problem-solving attitudes toward risk minimization [2]. To overcome feelings of helplessness and powerlessness, being able to do something constructive can help [2], and eco-anxiety has been shown to correlate not only with pro-environmental beliefs but also behaviors [1].

**Anxiogenic narratives**

Narratives have effects on the way public health threats are perceived as well as on the conviction that something can be done, “through processes of sense-making and creating shared convictions and desires” [5]. Beyond evidence on its impact, there are not yet any well-structured narratives on how to address climate change [5], leaving space for narratives of powerlessness and the inability to make a difference regarding climate change, which are certainly anxiogenic. Lots of research has been carried out on the effectiveness of fear communication to change behaviors, and the modest evidence available suggests that a threat has an effect only if its efficacy is high, that is, if one has the ability to negate the harm of the threat [10]. The same evidence suggests that under low efficacy, the effect of the threat is negative and could “cause people to engage in health-defeating behaviors” [10].

**Science versus politics**

Researchers should also be conscious of how their narratives are shaped by their own values and of the importance of maintaining a separation between politics, activism, and science because when science resembles politics, trust in evidence disappears [11]. Hence, when the authors of this international survey conclude that the “failure of governments to adequately address climate change and the impact on younger generations potentially constitutes moral injury” [3], they are not evidence-based but are formulating a moral and political opinion; they might be right, but that reveals their a priori beliefs about the question under study [4].

While science can identify solutions to public health problems, only politics can transform these solutions into reality [8,12]. Researchers might have a moral obligation to contribute to policymaking by providing evidence [9] but they have to accept that, in democratic jurisdictions, they are no more legitimate to make policy than any citizens. As scientists, they have to keep their axiological neutrality at least in their scientific productions to prevent a “white hat bias”, that is, the “distortion of research-based information in the service of what may be perceived as righteous ends” [13]. 

Researchers, and it is true for all domains of population health sciences, should carefully think about how societal and public health questions are framed, notably on how the severity of a threat is presented and what can be done to address this threat, because these narratives are determinants of anxiety or hope feelings in the population [5,6,10]; this is an important responsibility.

## Data Availability

Not applicable.
